# Benefits of WSES guidelines application for the management of intra-abdominal infections

**DOI:** 10.1186/s13017-015-0013-x

**Published:** 2015-03-18

**Authors:** Belinda De Simone, Federico Coccolini, Fausto Catena, Massimo Sartelli, Salomone Di Saverio, Rodolfo Catena, Antonio Tarasconi, Luca Ansaloni

**Affiliations:** Department of Emergency and Trauma Surgery, University Hospital of Parma, Via Gramsci 11, 43100 Parma, Italy; Department of General and Emergency Surgery, Papa Giovanni XIII Hospital, Bergamo, Italy; Department of Surgery, Macerata Hospital, Macerata, Italy; Department of Surgery, Maggiore Hospital of Bologna, Bologna, Italy; Oxford University, Oxford, Great Britain; Ospedali Civili di Brescia, Brescia, Italy

**Keywords:** Intra-abdominal infections, Antibiotics, WSES guidelines, Cost-effectiveness

## Abstract

**Introduction:**

The use of antibiotics is very high in the departments of Emergency and Trauma Surgery above all in the treatment of the intra-abdominal infections, to decrease morbidity and mortality rates; often the antimicrobial drugs are prescribed without a rationale and they are second-line antibiotics; this clinical practice increases costs without decreasing mortality.

Aim of our study is to report the results in the application to the clinical practice of the World Society Emergency Surgeons (WSES) guidelines for the management of intra-abdominal infections, at the department of Emergency and Trauma Surgery of the University Hospital of Parma (Italy) in 2012.

**Methods:**

A retrospective observational analysis was carried out about patients admitted in the department of Emergency and Trauma Surgery of Parma (Italy), between January 2011 and December 2012. The data are expressed as percentages (%) and means (± SD). The results of the compared groups were analyzed using the Pearson’s Chi-Square and Fisher’s tests. For means involving continuous numerical data, the independent sample T test and the Mann–Whitney U-test were used for normally and abnormally distributed data, respectively (the data had been previously tested for normality using the Kolmogorov-Smirnov test). A *p*-value < 0.05 was considered statistically significant.

**Results:**

Between January 2011 and December 2012, 2121 (968 in 2011 and 1153 in 2012) patients were admitted in the department of Emergency and Trauma Surgery (Italy) of Parma University Hospital with a diagnosis of acute IAI.

Morbidity in 2012 was 10,2% compared to 22.7% in 2011 and mortality in 2012 was 1,1% compared to 3,2% in 2011 (p < 0,05).

Costs for antibiotics in 2012 was 51392 euro, with a reduction of 31% compared to 2011.

**Conclusions:**

This study demonstrates that an inexpensive and easily application of guidelines based on medicine evidence in the use of antibiotics can lead to a significative reduction of hospital costs with outcomes improvement.

## Introduction

Antibiotics are the essential drugs that we have to fight and prevent bacterial infectious diseases. Improper and excessive use of antibiotics is the major worldwide problem because it has an important economic impact on increasing healthcare costs, caused by the selection of multi-drug resistant bacteria, resulting in a longer hospital stay and an higher mortality [1]. For the World Health Organization (WHO), the rational use of drugs requires that patients receive medications appropriate to their clinical needs, in doses that meet their own individual requirements, for an adequate period of time and at the lowest cost, to them and their community [2]; because each antibiotic has different unit dose of daily administration, a specific standardized method to evaluate the in-hospital antibiotic use was suggested and periodically update by WHO, the ATC/DDD index (Anatomical Therapeutic Chemical/Defined Daily Dose): it is considered the universal parameter to calculate the antibiotic use intensity [3]. Furthermore, the use of antibiotic prophylaxis, according to standardized protocols, has been shown to prevent post-surgical wound infections, which are the primary cause of morbidity and mortality in patients undergoing surgery.

The use of antibiotics is very high in the departments of Emergency and Trauma Surgery, above all in the treatment of the intra-abdominal infections (IAIs) to decrease morbidity and mortality rates. Often the antimicrobial drugs are prescribed without a rationale and they are second-line antibiotics; this clinical practice increases costs without decreasing mortality [4]. Sartelli et al., during the 1st Congress of the World Society of Emergency Surgeons (WSES), discussed in a multidisciplinary approach these problems, approving evidence based recommendations for the management of IAIs [1]. According to the WSES guidelines, the initial antibiotic therapy for IAIs is always empiric because the patient is often critically ill and microbiological data (culture and susceptibility results) usually take at least 48 hours to become fully available [1]. IAIs are classified as uncomplicated and complicated. The uncomplicated infections involve a single organ and do not spread to the peritoneum (antimicrobial therapy is indicated as first line approach); the complicated IAIs proceed beyond a single organ, causing localized or diffuse peritonitis and need for surgical and antimicrobial therapy.

IAIs are divided in 3 sub-groups: 1. community acquired extrabiliary infections: gastroduodenal perforations, small bowel perforations, acute appendicitis, acute diverticulitis, large bowel perforations; 2. community acquired biliary infections:acute cholecystitis, cholangitis; 3. hospital acquired infections: postoperative and non-postoperative peritonitis. Once the diagnosis of intra-abdominal infection is suspected, it is necessary to begin, as soon as possible, the empiric antimicrobial therapy, even if routine use of antimicrobial therapy is not appropriate for all patients with intra-abdominal infections. Source control should be obtained as early as possible after the diagnosis of postoperative intra-abdominal peritonitis has been confirmed [1].

The principles of empiric antibiotic treatment should be defined according to the most frequently isolated germs, always taking into consideration the local trend of antibiotic resistance. The choice of the antimicrobial regimen depends on the source of intra-abdominal infection, the risk factors for specific microorganisms, the resistance patterns and the clinical patient’s condition. In uncomplicated IAIs, when the focus of infection is treated effectively by surgical excision of the involved tissue, the administration of antibiotics is unnecessary beyond prophylaxis. In complicated IAIs, antimicrobial therapy is mandatory. Hospital acquired infections are commonly caused by larger and more resistant flora, and for these infections, complex multi-drug regimens are always recommended [1].

We report the results in the application to the clinical practice of the WSES guidelines for the management of intra-abdominal infections at the Department of Emergency and Trauma Surgery of Parma University Hospital (Italy) in 2012.

## Materials and methods

A retrospective observational analysis was carried out about patients with IAIs admitted to the Department of Emergency and Trauma Surgery of Parma University Hospital, between January 2011 and December 2012 The following parameters were collected: patients demographics, diagnosis, surgical procedures performed, antibiotic treatment, length of hospital stay (day) and outcomes. In 2011 and 2012, the same antibiotic drugs were available in our hospital, at the same price.

**Figure 1:**
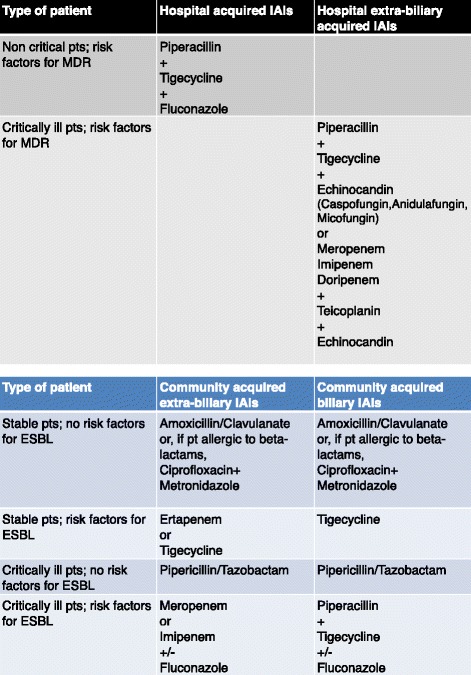
**WSES IAIs guidelines.**

In 2011, no guidelines were used, whereas in 2012 WSES IAIs guidelines were utilized. (Figure 1) Community acquired extra-biliary IAIs (gastro-duodenal perforations, small bowel perforations, acute appendicitis, acute diverticulitis, large bowel perforations) were treated with Ampicillin/Sulbactam or Ciprofloxacin (in patients with allergic reaction to Penicillin) +/−Metronidazole.

Community acquired biliary IAIs (cholecystitis, cholangitis) were treated with Ampicillin/Sulbactam or Ciprofloxacin, if allergic reaction to Penicillin, +/− Metronidazole, as first line therapy, if an ESBL or MDR pathogens were suspected.

Hospital acquired IAIs needed for a large spectrum therapy (high risk of ESBL or MDR pathogens involved) with Piperacillin/Tazobactam or Meropenem +/−Fluconazole +/− Tigecycline. Critically ill patients were often hospitalized in ICU.

All antibiotic treatments started with an i.v. administration followed by oral switch when appropriate (normal infection signs, normal infection laboratory parameters and resumption of oral feeding).

The data are expressed as percentages (%) and means (± SD). The results of the compared groups were analyzed using the Pearson’s Chi-Square and Fisher’s Exact tests, as appropriate, for proportions involving discrete data. The Fisher’s Exact test was used when the data were unequally distributed among the cells of the table, when the expected frequency of any cell was less than 5, or when the total number (N) was less than 50.

For means involving continuous numerical data, the independent sample T test and the Mann–Whitney U-test were used for normally and abnormally distributed data, respectively (the data had been previously tested for normality using the Kolmogorov-Smirnov test). A *p*-value < 0.05 was considered statistically significant.

## Results

Between January 2011 and December 2012, 2121 (968 in 2011 and 1153 in 2012) patients were admitted in the Department of Emergency and Trauma Surgery of Parma University Hospital with a diagnosis of acute IAI. The mean age was 58,8 years (SD ± 9,1) in 2011 and 59,1 in 2012 (SD ± 8.9); (p = n.s.). Male/ female ratio was 1,04 in 2012 and 1,02 in 2011 (p = n.s.). Complicated IAIs were 41,1% in 2012 and 38,7% in 2011. (p = n.s.).

**Figure 2:**
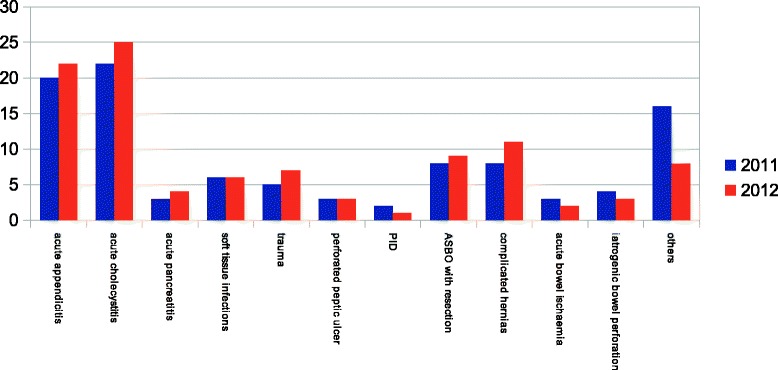
**IAI patients divided according to admission diagnosis in 2011 and in 2012.**

**Figure 3:**
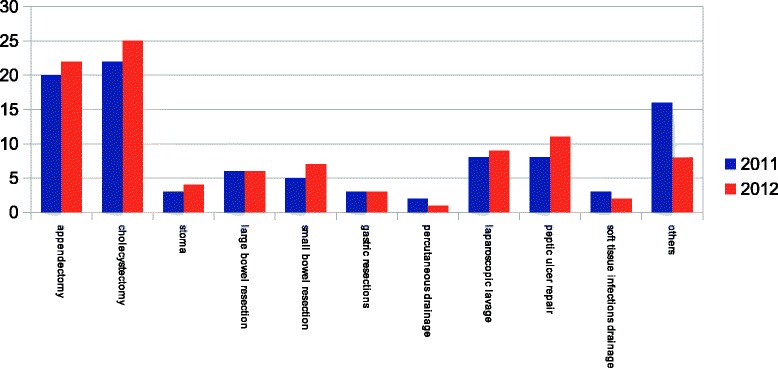
**Surgical procedures performed as source control on IAI patients in 2011 and in 2012.**

Empirical treatment was performed in 91,8% of patients in 2012 and in 95,3% of patients in 2011 (p = n.s.). In the Figure 2 patients were divided according to admission diagnosis: the majority of patients were affected by acute cholecystitis, followed by acute appendicitis and complicated abdominal hernias, including incisional hernias, without any statistical difference in distribution between 2011 and 2012. (p = n.s.). Surgical procedures, performed as source control on these patients, are shown in Figure 3: again there was not any statistical difference in distribution between 2011 and 2012. (p = n.s.).

All patients with IAIs were treated with antibiotics according to WSES guidelines.

**Figure 4:**
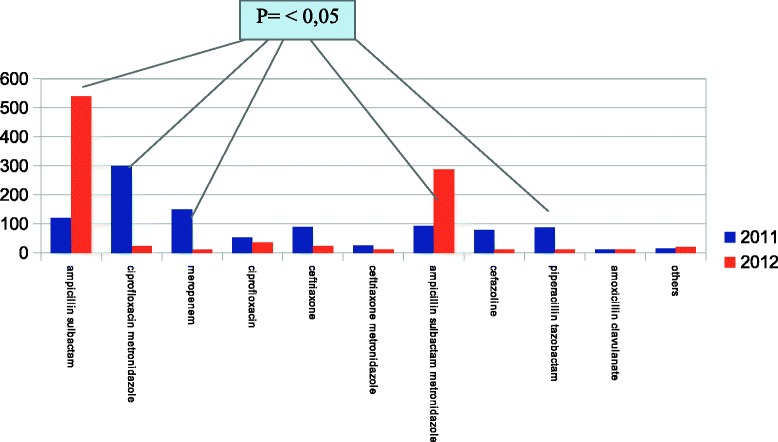
**The administrated antibiotic treatments in 2011 and in 2012.**

The administrated antibiotic treatments is shown in Figure 4. Between 2011 and 2012 there was a statistical significant difference for 5 antibiotic regimens: Ampicillin/Sulbactam, Ciprofloxacin plus Metronidazole, Meropenem, Ampicillin/Sulbactam plus Metronidazole, and Piperacillin/Tazobactam. The oral switch was performed in 540/ 968 (55,7%) patients in 2011 and in 691/1153 (59,9%) in 2012. (p = n.s.).

The mean length of intravenous therapy was 4.9 days (range 21–1) (DS 3,67) and the mean lenght of oral therapy was 3.23 days (range 13–3) (DS 3,18) in 2012, whereas the mean length of intravenous therapy was 5.4 days (range 33–2) (DS 4,22) and the mean lenght of oral therapy was 4,59 days (range 18–4) (DS 2,25); (p = n.s.) in 2011. Mean lenght of hospital stay was 7.5 days (range 43–1) (DS 6,08) in 2012 and 8,9 days (range 49–1) (DS 5,36) in 2011 (p = n.s.).

In-hospital mortality rate was 1.10% in 2012 vs 3.2% in 2011 (p < 0.05.) and morbidity was 10,2% in 2012 vs 22,7% in 2011 (p < 0.05). Costs for antibiotics in 2012 was 51392 euro compared to 75327 euro in 2011 (31,7% reduction). More common bacteria isolates were comparable between 2011 and 2012.

## Discussion

It’s worldwide accepted that a remarkable amount of antibiotics used in hospitalized patients is excessive or inappropriate; this irrational use of antibiotics leads to the emergence of drug resistant bacteria, associated with an higher rates of death, illness and prolonged hospital stay, with a considerable increasing of the healthcare costs. Besides the research involved with the development of new antibiotics has no progressed in parallel with the increasing rates of resistance, leaving clinicians with fewer options, often more expensive, for the treatment of some resistant infections.

In the recent literature, several studies argue on the necessity of the diffusion of valid guidelines, based on clinical evidence and on the bacterial resistance epidemiology, to rationalize the use of antibiotics [2-6]. Many authors highlight on the importance of the application of validated guidelines in clinical practice and of the surgical prophylaxis protocols, associated with adequate education programs for physicians and surgeons on the “diligent” prescription and administration of antibiotics, in reducing healthcare costs with considerable benefits in terms of cost-effectiveness [7-11].

In the present study, the application of WSES guidelines for the management of intra-abdominal infections was highly effective in reducing the number of unnecessary second-line antibiotics prescriptions and costs; it led to a 31% reduction of costs for antimicrobial drugs, keeping low morbidity and low mortality rates. The source control associated with an adequate antimicrobial therapy are efficacy to decrease morbidity and mortality rates.

This study demonstrates that an inexpensive and easily application of guidelines based on medicine evidence in the use of antibiotics can lead to a significant reduction of hospital costs. There is an urgent need to develop education programs, to spread valid guidelines in the use of antimicrobial agents, to limit the emergence of bacterial resistance, responsible of the increasing in the incidence of “difficult” infectious diseases and deaths, and to reduce costs resulting from this global problem [10-12].
